# The Prevalence of Behavioral and Emotional Problems and Their Associated Factors Among Children and Adolescents in Jordan: Findings from a National School-Based Survey

**DOI:** 10.3390/pediatric16040103

**Published:** 2024-12-19

**Authors:** Bayan Labib, Yousef Khader, Sara Abu Khudair, Mohannad Al Nsour, Eizaburo Tanaka

**Affiliations:** 1 Faculty of Dentistry, Al-Ahliyya Amman University, Amman 19328, Jordan; 2 Department of Community Medicine, Public Health and Family Medicine, Faculty of Medicine, Jordan University of Science and Technology, Irbid 22110, Jordan; yskhader@just.edu.jo; 3 Health Sciences Unit, Faculty of Social Sciences, Tampere University, Tampere 33520, Finland; sara.a.khudair@gmail.com; 4 The Eastern Mediterranean Public Health Network, Amman 11196, Jordan; executive.director@emphnet.net; 5 College of Arts and Sciences, Komaba Organization for Educational Excellence, The University of Tokyo, Tokyo 113-0033, Japan; eizaburo.tanaka.20@alumni.ucl.ac.uk

**Keywords:** mental health, emotional problems, behavioral problems, schoolchildren, adolescents, Jordan

## Abstract

Background: Global research has reported that the number of children and adolescents suffering from mental health issues has increased over the past decades. In Jordan, there has been a growing interest in investigating mental health among these groups in the most recent decade; nevertheless, only a few studies have covered behavioral and emotional problems. This study aimed to estimate the prevalence of behavioral and emotional problems among children and adolescents in Jordan and investigate their associated factors. Methods: A large-scale, national, school-based cross-sectional study was conducted between December 2022 and April 2023 on children and adolescents living in Jordan aged between 8 and 18 years. The study included public schools, private schools, UNRWAs schools, Zaatrai camp schools, and non-formal education centers. The Strengths and Difficulties Questionnaire was used to measure behavioral and emotional problems. Results: About 13.9% of the children had abnormal difficulty scores, and they suffered the most from emotional symptoms (17.9%). Syrian children in refugee camps had the highest rate of total difficulty (19.3%). In the adolescents, 19.7% had high levels of total difficulty, where conduct problems were the most reported (17.6%), and Syrian adolescents in refugee camps were highly affected (22.2%). The number of traumatic events, physical activity, problematic internet use, and family affluence were significantly associated with an increased risk of having behavioral and emotional problems in both the children and adolescents. Conclusions: A significant proportion of children and adolescents struggle with emotional and behavioral problems in Jordan, and serious efforts are needed to enhance the status of mental health for adolescents and children.

## 1. Introduction

Mental health is a major component of and a vital prerequisite for, along with physical and social health, establishing a complete status of health [1]. Mental health issues in childhood and adolescence can significantly affect concurrent achievements, such as academic performance, as well as later occupational and family lives [2,3]. Mental health problems in children and adolescents is a broad term that includes several mental disorders such as depression, anxiety, behavioral and emotional problems, attention-deficit hyperactivity disorder, and many others. Global research has reported that the number of children and adolescents suffering from anxiety disorders, major depressive disorders, and conduct disorders has increased over the past 30 years [4]. 

In terms of behavioral and emotional problems, UNICEF reported in 2019 that one in seven adolescents had mental health problems, with conduct disorders making up 20.1% of these problems [5]. In the UK, a recent study that included 28,160 adolescents and used the Strengths and Difficulties Questionnaire (SDQ) to assess mental health status showed that 2 in 5 adolescents scored above the borderlines for emotional problems, conduct problems, or hyperactivity [6]. The study also found that factors such as gender, poverty, child-in-need status, ethnicity, and age were all associated with increased odds of having mental health problems. Moreover, a national survey in China that targeted schoolchildren and adolescents reported that 17.6% had behavioral and emotional problems [7]. Observations from India (with a total difficulty score of 17.2% using the SDQ) and Sri Lanka (13.8%) have also shown alarming estimates of behavioral and emotional difficulties among children and adolescents [8,9]. In a study in Croatia, 10.4% of adolescents had borderline or abnormal scores on the total difficulty score using the SDQ, where girls scored higher on the total, emotional, and hyperactivity scales and boys scored higher on the conduct and peer relationship scales [10]. A recent study in Afghanistan was conducted on schoolchildren (grades 5–10) using the SDQ and reported the total difficulty score to be 5.7%, where gender, grade, worrying about food shortage, and concern about losing a house significantly predicted the self-reported total difficulty scores [11]. Factors like poverty, an unhealthy lifestyle (including smoking, a lack of fruit and vegetable intake, and a sedentary lifestyle), and also exposure to different traumatic events, such as COVID-19 and the loss of a beloved family member, can all affect the status of mental health [12,13].

In Jordan, there has been a growing interest in investigating the different aspects of mental health in the most recent decade [14]. Nevertheless, only three studies have been found to concentrate specifically on behavioral and emotional problems among children and adolescents in Jordan. The first study reported the rate of behavioral and emotional problems to be 11.7% but was limited to Amman city, and only adolescents were examined [2]. It demonstrated the relationship between two factors, gender and grade point average (GPA), and behavioral and emotional problems. Refugees, who constitute a considerable percentage of the population in Jordan, were not a target population for the aforementioned study. On the contrary, the second study dealt with Jordanians and Syrian refugees and assessed the behavioral and emotional problems of schoolchildren in four different cities in Jordan [15]. Total difficulties and peer relationship problems exceeded the normal values in more than half of the children, with the emphasis that Syrian children were more likely to develop behavioral and mental difficulties. The third study assessed behavioral problems but considered adolescents residing in institutional care and found a high level of mental health problems [16]. Our study investigated behavioral and emotional problems in a more comprehensive manner in terms of age, which included a younger age category (8–11 years old) that was not covered in the previous studies. It also covered a wider geographical area in Jordan (north, middle, and south) with an increased sample size compared to the previous studies. Moreover, this study examined many factors along with demographic characteristics such as eating habits, physical activity, smoking status, previous exposure to traumatic events, and problematic internet use.

Due to the fundamental role mental wellbeing plays in the overall health of children and adolescents and due to the limitations of the regions, age groups, and variables assessed in the previously mentioned studies in Jordan, the aim of this study was to estimate the prevalence of behavioral and emotional problems among children and adolescents in Jordan and investigate their associated factors.

## 2. Materials and Methods

### 2.1. Study Design and Population

A large-scale, national, school-based cross-sectional design was utilized to conduct this study among children and adolescents living in Jordan. The target population of this study was Jordanian children and adolescents, as well as those of other nationalities such as Syrian and Palestinian refugees aged between 8 and 18 years. The study included public schools, private schools, UNRWAs schools, Zaatrai camp schools, and non-formal educations centers. All grades from 3 to 12 were included in this study.

### 2.2. Sample Size

In terms of sample size, this study utilized an equation that relied on prevalence, the confidence interval, and precision (half the confidence interval’s width) [17]. Based on what was found in the literature, the prevalence of common mental problems was assumed to be 25% [5]. A confidence level of 95% and a confidence interval width of 4% (precision 2%) were selected. The formula yielded the required sample size of 1081 participants. The sample size was then adjusted by a design effect of 2 and 70% complete responses (a 30% loss). To have adequate power to conduct subgroup analysis, the number of potential participants invited to this survey was increased to 9000 children and adolescents.

### 2.3. Sampling and Setting

The participants in this study were recruited using a multi-stage stratified cluster sampling technique in order to have a nationally representative sample. Schools in Jordan are generally classified into three categories according to the governing authorities: public, private, and United Nations Relief and Work Agency (UNWRA). Public and private schools, managed by Jordan’s Ministry of Education (MoE), primarily serve Jordanian citizens along with some non-Jordanians. Private schools offer advanced facilities such as laboratories, libraries, and extracurricular programs. Teachers across these schools are MoE-trained, typically holding bachelor’s degrees in education or related fields. UNRWA operates schools specifically for Palestinian refugees in Jordan, located in both refugee camps and urban areas. To meet demand, some of these schools adopt a double-shift system, with morning and afternoon sessions. Teachers, predominantly Palestinian refugees, are hired and trained by UNRWA to deliver education tailored to this community. In the Zaatari camp, schools are dedicated to Syrian refugees and often face challenges of overcrowding due to the high refugee population. Funded by international organizations and donor governments, these schools follow Jordan’s MoE curriculum. The teaching staff comprises a mix of MoE-trained Jordanian teachers and Syrian refugees who receive training to deliver the curriculum effectively.

This study aimed to include all school categories; therefore, the first-level stratification of the school population was based on their governing authority (public, private, and UNWRA). These were divided into governorates (12 areas). The public schools were then stratified according to refugee context into two levels: regular (first morning shift for Jordanians) and second shift (afternoon shift for Syrian refugees). The public schools were further stratified by gender (boys, girls, and mixed schools). 

The researchers obtained a list of all schools from the Jordanian Ministry of Education, and they ordered the schools according to the number of students. Subjects were then recruited through systematic sampling for each stratum. One replacement school was identified for each school at the time of sample selection. Contact with replacements was only made if the selected school declined participation. For each selected school, the following were considered: all classes from grades 3 to 12 were included, and 30% of students in each class were chosen by systematic sampling (to increase the number of participating schools). It should be noted that the number of students chosen was proportional to the number of students in each governing authority (self-weighted sample). This study also considered those who were not attending or dropped out of school by reaching non-formal education centers. Non-Formal Education (NFE) is a Ministry of Education (MoE)-certified program designed for adolescents aged 13 and older who have been out of school for three years or more, making them ineligible for formal education. The Drop-Out Program provides a two-year learning cycle, after which graduates receive a certificate equivalent to a 10th-grade qualification. This certification enables access to vocational-technical education, homeschooling to prepare for high school exams, or entry into work and business opportunities. Currently, there are approximately 118 UNICEF-supported NFE Drop-Out centers nationwide, operated in collaboration with UNICEF’s partners. The curriculum is taught by Ministry of Education teachers within public schools. Data were collected from northern and central Jordan, as the majority of non-formal educational program attendees were located in this region.

### 2.4. Data Collection

Trained data collectors reached out to potential subjects in their selected schools and non-formal education centers in the period between December 2022 and April 2023. Participants from 7th grade to 12th grade filled out a questionnaire by themselves under the supervision of the data collectors, while grade 3–6 students handed the questionnaire to their caregivers.

Before collecting the sample, the data collectors received extensive training in different aspects of the process of data collection. This included training on ethical consideration, confidentiality, how to communicate professionally with the study sample, how to provide clear information about the aim of the study, and how to achieve voluntary participation and anonymity. During data collection for students in 7th–12th grade, the data collectors kept enough space between the students to reduce social desirability bias and ensure genuine responses. Before visiting the schools and non-formal educational centers, the data collectors contacted principals in advance to arrange for a data collection appointment.

### 2.5. Study Instruments

The survey for this national study included multiple tools to measure a variety of mental health problems among children and adolescents and their associated factors in Jordan. In this paper, we are specifically concerned about behavioral and emotional problems and their related factors. Therefore, only the instruments that were related to measuring behavioral and emotional problems and their possible associations will be described in this section.

Firstly, participants were asked about their demographic characteristics, health status, and socio-economic characteristics (SES). This included the following: gender, age, school classification (public, private, UNRWA, non-formal education, Al-Zaatari camp), school type (boys, girls, mixed), region of residence, nationality, parental status (educational level, living together or separated, work), income (source, total income), and medical history (COVID-19, medications on a regular basis, a history of parents’ mental health problems). In addition, the SES was evaluated by the Family Affluence Scale (FAS)-III [18]. The answers of the participants on the FAS were summed and ranged from 0 to 13, and the following cutoff values were used to categorize the results: 0–7 (low), 8–11 (medium), and 12–13 (high).

Secondly, the Strengths and Difficulties Questionnaire (SDQ) was used to assess behavioral and emotional problems, the dependent variable [19]. The SDQ required participants to rate 25 statements on a 3-point Likert scale (not true, somewhat true, and certainly true), and the scale included five subscales (5 items each) which were as follows: emotional symptoms, conduct problems, hyperactivity or inattention, peer relationship problems, and prosocial behavior. Each scale ranged from 0 to 10, and the sum of the 4 subscales (excluding prosocial behavior) gave a total difficulty score. The total difficulty score was then categorized as normal or abnormal. The cutoff values for defining the abnormal attributes of the total difficulty score were 17–40 for parents’ versions and 20–40 for students’ versions. Subscale scores were only calculated if at least three to five questions were answered, and if fewer than three were completed, the subscale score was treated as missing.

Thirdly, independent variables regarding lifestyle, including eating habits, physical activity, smoking, and the number of traumatic events, were assessed. Eating habits included the following two factors: fruit intake per day (0, 1–2, ≥3) and vegetable intake per day (0, 1–2, ≥3). In terms of physical activity, it included one question that examined the frequency of weekly engagement in light physical activity for at least 60 min per day (0, 1–4, ≥5). Also, smoking was assessed with two answers: yes (cigarettes or water pipes) or no. In addition, the number of traumatic events that the participants were exposed to in their lives was assessed by asking about 18 potential traumatic events with two answers (happened to me, did not happen to me) based on the Revised Adverse Childhood Experiences (ACEs) Questionnaire and the Life Events Checklist for DSM-5 (LEC-5) [20].

Finally, problematic internet use, which was measured by the Problematic Internet Use Questionnaire Short Form (PIUQ-SF-6) was examined [21]. The questionnaire is a 5-point Likert scale of six items that evaluates the misuse of the internet (obsession, neglect, and control disorder). Scores ranged from 6 to 30, and a cut-off score of higher than 15 was used to classify results into two categories: at risk of problematic internet use and not at risk.

The selected instruments for this study are internationally recognized and validated in English. Permissions were obtained to use the instruments from the developers, or the ones who own the copyright, while others are available in the public domain. As for translation to Arabic, a forward–backward method was utilized where two bilingual forward translators, two bilingual backward translators, two clinical health scientists, and one epidemiologist participated and reviewed the translated Arabic version. There were two versions of the survey: a self-reported version for students in grades 7–12 and a parent’s version for students in grades 3–6. The non-formal education students were asked to complete the self-reported version because they were 12 years of age or older.

### 2.6. Pilot Study

A pilot study was conducted on a total of 60 students in two schools in northern Jordan on 7 November 2022. Thirty students were selected from a girls’ school in Irbid, and another thirty were selected from a boys’ school in the Al-Zaatari camp in Mafraq. In each school, 15 surveys were handed out to students in grades 7–12, and the other 15 were given to the parents of students in grades 3–6. The study helped the researchers assess readability, clarity, and the time needed to fill out the questionnaire and identify logistical problems. The changes identified in the piloting phase were reflected in the parent and student versions in both languages.

### 2.7. Statistical Analysis Plan

Data analysis was carried out using IBM SPSS 24 (IBM Corp. Released 2016. IBM SPSS Statistics for Windows, Version 24.0 Armonk, NY, USA: IBM Corp.) Descriptive statistics including counts and percentages for categorical variables were used to demonstrate the characteristics of the study sample and to investigate the prevalence of behavioral and emotional problems. Inferential statistics were applied by performing binary logistic regression analysis in order to investigate the association between behavioral and emotional problems and a variety of independent factors including gender, nationality, region, family affluence, fruit intake, vegetable intake, light physical activity, smoking, the number of traumatic events, and problematic internet use. Throughout the inferential statistical analysis of this study, any *p* value of 0.05 or less was considered statistically significant.

### 2.8. Ethical Consideration

Ethical approvals were obtained from the Institutional Research Committees at Jordan University of Science and Technology (JUST) and the Jordan Ministry of Health. This study followed the Declaration of Helsinki and other local ethical guidelines for medical and health research involving human subjects. Each participant (adolescent or caregiver) was provided a copy of the informed consent form, including information about the aim of the study, benefits, potential risks, if any, and the voluntariness of their participation, assuring the confidentiality of the data collected and participants’ anonymity and explaining that they have the right to refuse to answer any of the study questions without any consequences or to withdraw at any time without explanation. Written informed consent from the caregivers of the participants and informed consent from the participants were obtained before data collection. On the same note, all the gathered data were stored in a safe, locked cabinet with only limited access by the study team.

## 3. Results

### 3.1. Participants’ Socio-Demographic Characteristics

This study included a total of 8000 participants, where there were 3593 children (8–11 years) and 4407 adolescents (12–18 years). The majority of the children went to public schools (64.7%), as did the majority of the adolescents (64.8%). Males represented 41% and 44.5% of the children and adolescents, respectively. The majority of the adolescents were Jordanians (66.2%), and just over half of the adolescents (51.1%) reported that they live in the middle region. In the children, more than half were Jordanians, and 46.8% of the children lived in the north region. About half of the participants (53.2% of children and 55.5% of adolescents) fell within the medium family affluence scale. Table 1 presents a summary of the socio-demographic characteristics of the study’s participants, both the children and adolescents.

### 3.2. Prevalence of Behavioral and Emotional Problems in Children and Adolescents

The total difficulty score was abnormal in 13.9% of children. The children suffered the most from emotional symptoms (17.9%). This was followed by conduct problems (16.1%) and peer problems (16.0%). Prosocial behavior, which is a positive trait, was only prevalent among 4.3% of the children. When it came to gender, of all emotional and behavioral problems, conduct problems were the most prevalent among males (22.0%) and emotional problems were the most prevalent among females (17.8%). Table 2 shows the prevalence of behavioral and emotional problems among the children by gender.

When it came to adolescents, the total difficulty score was abnormal in 19.7% of participants. Conduct problems (17.6%), followed by peer problems (16.3%), were the highest among the adolescents. Both male and female adolescents had conduct problems as the most prevalent problem in their emotional and behavioral problems. Only 11.2% of the adolescents showed prosocial behavior. Table 3 shows the behavioral and emotional problems among the adolescents by gender.

### 3.3. Prevalence of Behavioral and Emotional Problems According to Nationality

Among the children in this study, the Syrian camp refugees had the highest rate in their total difficulty score (19.3%). The Syrian child camp refugees also showed the highest rates on most subscales including abnormal emotional problems (25.7%), conduct problems (18.0%), and hyperactivity (12.7%). Children who were Jordanians, Palestinian camp refugees, and Syrians living outside camps showed a lesser prevalence of total difficulties scores at 12.5%, 12.9%, and 13.9%, respectively. Table 4 presents the behavioral and emotional symptoms among the children according to nationality.

As for the adolescents, Syrians living in a camp had a higher prevalence of emotional and behavioral problems, at 22.2%, compared to Jordanians, Syrians residing outside camps, and Palestinian camp refugees. Notably, the Jordanians had the highest rates of emotional problems, at 14.3%, and hyperactivity, at 12.7%, while the Syrian camp refugees had the highest conduct problems (22.2%). Prosocial behavior was mostly prevalent among the Syrians living outside camps. Table 5 demonstrates the behavioral and emotional problems among the adolescents according to nationality.

### 3.4. Factors Associated with Total Difficulty Score Among Children

Binary logistic regression analysis indicated that in the children, males were significantly more likely to have emotional and behavioral problems than females (OR =1.445; *p* = 0.002). Also, Syrians and Palestinians living in camps were more likely to suffer from total difficulties, with odds ratio of 1.811 and 1.211, respectively, compared to Jordanians. In addition, the more children were exposed to traumatic events, the more they would have emotional and behavioral problems. Children and adolescents can face a wide range of traumatic events that can impact their mental health. These traumatic events range from being infected or having a family member infected with COVID-19, not having enough to eat or having to wear old clothes, failing an exam, and the sudden accidental death of any family member. Children who were at risk of problematic internet use were 1.98 times more likely to have behavioral and emotional problems. Other factors such as region, fruit intake per day, vegetable intake per day, light physical activity per week, smoking, and family affluence affected the scores of the total difficulties. Table 6 shows the results of the binary logistic regression analysis of the total difficulty score and associated factors among the children.

### 3.5. Factors Associated with Total Difficulty Score Among Adolescents

In the adolescents, males were less likely to have emotional and behavioral problems than females (OD = 0.87). Adolescents who lived in the middle and south regions were more likely to suffer from difficulties than those living in the north, with odds ratios of 1.307 and 1.376, respectively. Notably, adolescents with no intake of fruit or vegetables per day were significantly more likely to experience emotional and behavioral problems than those having three or more servings of fruit or vegetables per day. In addition, adolescents who were at risk of developing problematic internet use were 2.084 more likely to have emotional and behavioral problems. Table 7 summarizes the factors associated with the total difficulty score among the adolescents.

## 4. Discussion

This study showed that the prevalence of emotional and behavioral problems for all SDQ domains was 13.9% in the children and 19.7% in the adolescents in Jordan. In the children, emotional problems were the most common (17.9%), followed by conduct problems (16.1%) and then peer problems (16%). Meanwhile, in the adolescents, emotional problems were the third most common, with a prevalence rate of 14.1%, and they were preceded by higher rates of peer problems (16.3%) and conduct problems (17.6%). Hyperactivity was the least common emotional and behavioral problem in both the children and adolescents.

Syrian child camp refugees showed the highest overall difficulty percentage (19.3%) compared to other nationalities (less than 14%) including Palestinian camp refugees (12.9%).

Palestinian refugees have been settled in Jordan for decades, allowing for more stable living conditions and integration into society. This stability can contribute to better mental health and coping mechanisms. In contrast, Syrian refugees, many of whom arrived during or after the Syrian conflict, often face ongoing instability and uncertainty, which could lead to more psychological distress. Syrian child refugees may have experienced more recent and severe trauma, including witnessing violence, displacement, and loss during the Syrian conflict. This acute exposure can result in higher emotional and behavioral difficulties compared to Palestinian children, many of whom have not experienced the same level of recent conflict. In Jordan, a previous study compared Jordanian schoolchildren and Syrian refugees, where the Syrian refugees scored higher in the total difficulty score, and the study did not include a comparison between Syrian and Palestinian refugees [15]. Therefore, further research is needed for this. In the adolescents, all nationalities, including Jordanians, Syrians, and Palestinians, had similarly increased rates of behavioral and emotional problems.

It was observed that in the children, being male, being Syrian and living in a refugee camp, being exposed to three or more traumatic events, not practicing any light physical activity, using the internet in a problematic way, and having low family affluence significantly increased the odds of having total difficulties. In the adolescents, exposure to four or more traumatic events, not having a fruit or vegetable intake, not engaging in any light physical activity, problematic internet use, smoking, and having sub-high family affluence significantly increased the odds of having emotional and behavioral problems.

The overall difficulty prevalence rates observed among the adolescents in this study were far below those reported previously in Jordan, which were 52.5% among Jordanians and 58.2% among Syrians using the SDQ instrument [15]. A possible explanation of this might be that the previous study covered some governorates and was not at a national level. Nevertheless, both studies were consistent in revealing that conduct problems and peer problems were the most common emotional and behavioral problems. Another study in Jordan also used the SDQ to assess emotional and behavioral difficulties among adolescents and showed relatively lower prevalence rates for overall difficulties and a difference in domains’ ranking when compared to our results [2]. The aforementioned study was limited to Jordanian adolescents, and data collection was limited to only two schools in Amman, which may be the explanation for this inconsistency with our study. The findings reported from Jordan, despite timing—before or after COVID-19—and despite governorates, suggest that emotional and behavioral problems are prevalent among Jordanian, Palestinian, and Syrian adolescents and children and that these problems are enduring issues that need to be targeted by effective interventions for a better mental health status in these age groups.

Conduct problems, according to our study, were the most common among the adolescents, with a percentage of 17.6%. This percentage accords with observations from two other studies that used the SDQ and reported conduct problems at rates of 18.5% and 15.2% in the UK and India, respectively [6,9]. Conduct problems include misbehaviors like starting fights with others unreasonably, cheating, lying, stealing, and rule-breaking, and they can be looked at as one of the consequences of missing or incomplete guidance for adolescents from an early age. For conduct problems, it is recommended to use validated programs for children and adolescents with components targeting young persons, parents, teachers, and the environment [22]. For young person, intervention must focus on social, cognitive, and emotional skill development; for parents, it must focus on training in positive discipline, child anger management, and communication; and for teachers, it must focus on training in cooperative teaching, conflict resolution, proactive management, positive reinforcement, and finally improving the school environment [22]. Early upbringing is also very important as strict or unassertive parenting can both be unwise for upbringing children and, rather, a balanced style of parenting is required.

In terms of peer problems, which indicate difficulty establishing and maintaining positive relationships with peers, suggesting problems with communication and interpersonal skills, our study supports the high prevalence of peer problems reported by a previous Jordanian study [15]. These findings from Jordan are higher than those reported in the UK and India, where they were reported to be 7.3% and 5.6%, respectively [6,9]. This highlights the necessity in Jordan providing solutions for better and healthier social communication for children and adolescents to overcome this potential obstacle to their mental health. Schools can play a fundamental role in enhancing mental health among children and adolescents. Schools can adopt specific and concrete measures that go beyond general support, such as implementing school-based mental health programs. These programs can offer regular counseling services, create peer support groups, and train teachers to recognize early signs of mental health problems including emotional and behavioral problems. The integration of mental health education into formal curricula would be beneficial to promote awareness and resilience among students.

Our study showed that the overall difficulty score increased significantly when the children and adolescents were exposed to multiple traumatic events. Events like COVID-19 and the Syrian conflict were expected and reported in this study population. Such traumatic events led to the loss of beloved lives, the loss of jobs and residences, or food insecurity, which in turn had a negative impact on mental health [12]. In addition, our study revealed that unhealthy lifestyles, where there was physical inactivity and problematic use of the internet, significantly increased the odds of having emotional and behavioral problems among the children and adolescents. People in the present day, with revolutionary and easy access to the internet through phones and all the available services and entertainment offered online, are very much attached to their mobile phones, which can lead with other possible factors to adopting a sedentary lifestyle. This sedentary lifestyle can limit the chance to be socially and physically active, which can be responsible in part for establishing some emotional and behavioral problems. Other unhealthy lifestyle practices, such as smoking and a lack of fruit and vegetable intake per day, also significantly increased the odds of overall difficulty among the adolescents in this study.

This study showed that the adolescents who had zero intake of fruit and vegetables were more likely to have behavioral and emotional problems compared to those who had three or more servings of fruit and vegetables per day. This relationship might be influenced or explained by other factors such as socio-economic status, lifestyle habits, or any other psychological factors; therefore, further research is needed to explore additional factors. In Jordan, it has been reported that less than one-third of adolescents eat fruit and vegetables daily, and it has also been reported that in Arab countries only 10–29% of adolescents meet the WHO recommendation regarding having five servings of fruits and vegetables per day [13,23,24]. Adolescents, in general, do not pay much attention to what they should eat to achieve a healthy eating style. They are young and generally healthy, and they like to try different foods and drinks without paying much attention to their nutritional value. Some other adolescents might undergo extreme dieting to look fit and go along with what they think are trendy looks and perfect body shapes, which in turn can have devastating impacts on their overall health. Therefore, healthy dieting should be considered while addressing the issue of emotional and behavioral problems, as poor mental health and an inadequate diet were found to be related.

Finally, this study showed that in both the adolescents and children, the low financial status of families significantly played a role in increasing the odds of having emotional and behavioral problems. Social and economic conditions in which individuals are raised and grown are believed to impact their mental health [25]. In Scotland, it was reported that people living in the most deprived areas showed higher levels of mental problems (23% of men and 26% of women) than those living in the most affluent places (12% of men and 16% of women) [26].

This study has a few limitations. It is a cross-sectional study that relied on surveys to collect data, thus having the limitation that surveys in general have where participants could under- or over-report their given answers. Data regarding children (8–11 years old) were collected through their caregivers, and they may have had a tendency not to point out the mental issues or abnormalities that their children may suffer from.

## 5. Conclusions

This study aimed to assess the prevalence of emotional and behavioral problems and their associated factors among children and adolescents in Jordan. A significant proportion of the children and adolescents struggled with emotional and behavioral problems, despite their nationalities. Emotional problems, conduct problems, and peer problems seem to be urgent issues for children and adolescents to be addressed. Factors including the number of traumatic events, physical inactivity, lack of fruit and vegetable intake, problematic internet use, and family affluence significantly increased the chance of having emotional and behavioral difficulties among the children and adolescents.

This study highlights the importance of efforts needed to enhance the status of mental health for adolescents and children in Jordan. A reasonable approach to tackling emotional and behavioral problems is to assure that a healthy environment is provided for children from an early age throughout their upbringing. This plays a fundamental role in their behaviors and emotions later in their lives. Educational and training programs that target parents, teachers, and those taking roles in raising children and cover the fundamentals of healthy upbringing are recommended. In addition, providing children with a positive environment to socially communicate with one another is of value. Extracurricular activities such as sport classes or artistic classes can help children communicate in a productive way. This study also suggests the need for action plans to identify and manage children and adolescents who have been exposed to traumatic events and suffer from their negative consequences. It is also recommended to encourage healthy lifestyles among children and adolescents where more physical activity, healthy eating, and smoking avoidance are adopted.

## Figures and Tables

**Table 1:** The socio-demographic characteristics of the 8000 children and adolescents.

Variable	Children (8–11 Years) n = 3593		Adolescents (12–18 Years) n = 4407		Total N = 8000	
	n	%	n	%	N	%
School classification						
Public school	2325	64.7	2811	63.8	5136	64.2
Private school	330	9.2	717	16.3	1047	13.1
UNRWA school	459	12.8	343	7.8	802	10.0
Non-formal education center	2	0.1	86	1.9	88	1.1
Zaatrai Camp school	477	13.2	450	10.2	927	11.6
School type						
Male	1058	29.4	1716	38.9	2774	34.7
Female	1003	28.0	1801	40.9	2804	35.0
Mixed	1532	42.6	890	20.2	2422	30.3
Gender						
Male	1474	41.0	1959	44.5	3433	42.9
Female	2119	59.0	2448	55.5	4567	57.1
Region						
Middle	1422	39.6	2254	51.1	3676	46.0
North	1682	46.8	1704	38.7	3386	42.3
South	489	13.6	449	10.2	938	11.7
Nationality						
Jordanian	2078	57.8	2917	66.2	4995	62.5
Syrian living in a camp	549	15.3	510	11.6	1059	13.2
Syrian living outside camps	871	24.3	739	16.8	1610	20.1
Palestinian living in a camp	94	2.6	76	1.7	170	2.1
Other	1	0.0	165	3.7	166	2.1
Parents’ marital status						
Living together	3323	92.5	3938	89.3	7261	90.7
Separated	158	4.4	233	5.3	391	4.9
One of them or both are deceased	112	3.1	236	5.4	348	4.4
Mother’s education						
A diploma or higher education	1221	34.0	1666	37.8	2887	36.1
Less than a diploma	2372	66.0	2741	62.2	5113	63.9
Father’s education						
A diploma or higher education	1015	28.2	1630	37.0	2645	33.1
Less than a diploma	2578	71.8	2777	63.0	5355	66.9
Father works	2733	76.1	3465	78.6	6198	77.5
Mother works	709	19.7	1101	25.0	1810	22.6
Family income source						
The work of one or both parents	2746	76.4	3732	84.7	6478	80.9
Humanitarian organizations	340	9.5	212	4.8	552	6.9
Relatives and friends	70	1.9	80	1.8	150	1.9
Has no income	437	12.2	383	8.7	820	10.3
Monthly income (JD)						
Less than 300	1835	51.1	1725	39.1	3560	44.5
301 to 500	1146	31.9	1391	31.6	2537	31.7
>500	612	17.0	1291	29.3	1903	23.8
Family Affluence						
Low affluence	1376	38.3	723	16.4	2099	26.2
Medium affluence	1912	53.2	2446	55.5	4358	54.5
High affluence	305	8.5	1238	28.1	1543	19.3
Ever had COVID-19						
Yes, confirmed by a positive test or based on medical advice	526	14.6	1247	28.3	1773	22.2
Unsure	759	21.2	1028	23.3	1787	22.3
No	2308	64.2	2132	48.4	4440	55.5
Takes medications regularly	134	3.7	396	9.0	530	6.6
Has a family member(s) with psychological issue(s)	160	4.5	242	5.5	402	5.0
JOD = Jordanian Dinar						

**Table 2:** The behavioral and emotional problems among children by gender.

Subscales/Scale	Children (8–11 Years) n = 3579						
	Male n = 1466		Female n = 2113		Total N = 3579		*p*-Value
	n	%	n	%	n	%	
Emotional symptoms	265	18.1	377	17.8	642	17.9	0.857
Conduct problems	323	22.0	254	12.0	577	16.1	0.000 *
Hyperactivity	200	13.6	157	7.4	357	10.0	0.000 *
Peer problems	262	17.9	312	14.8	574	16.0	0.013 *
Prosocial behavior	77	5.3	74	3.5	151	4.2	0.010 *
Total difficulties ^1^	257	17.5	240	11.4	497	13.9	0.000 *

^1^ The total difficulties scale score was generated by summing scores from all the subscales except the prosocial scale. Prosocial behavior is a positive trait. * *p* value is significant at 0.05 or less.

**Table 3:** The behavioral and emotional problems among the adolescents by gender.

Subscales/Scale	Adolescents (12–18 Years) n = 4400						
	Male n = 1954		Female n = 2446		Total N = 4400		*p*-Value
	n	%	n	%	n	%	
Emotional symptoms	240	12.3	381	15.6	621	14.1	0.002 *
Conduct problems	371	19.0	404	16.5	775	17.6	0.033 *
Hyperactivity	208	10.6	332	13.6	540	12.3	0.003 *
Peer problems	318	16.3	397	16.2	715	16.3	0.969
Prosocial behavior	255	13.1	237	9.7	492	11.2	0.000 *
Total difficulties ^1^	358	18.3	508	20.8	866	19.7	0.042 *

^1^ The total difficulties scale score was generated by summing scores from all the subscales except the prosocial scale. Prosocial behavior is a positive trait. * *p* value is significant at 0.05 or less.

**Table 4:** Behavioral and emotional symptoms among children **according** to nationality.

Subscale/ Scale	Children (8–11 Years) N = 3579								
	Jordanian n = 2074		Syrian Camp Refugees n = 544		Syrian Urban Refugees n = 868		Palestinian Camp Refugees n = 93		*p* Value
	n	%	n	%	n	%	n	%	
Emotional problems	300	14.5	140	25.7	187	21.5	15	16.1	0.000 *
Conduct problems	340	16.4	98	18.0	124	14.3	15	16.1	0.294
Hyperactivity	212	10.2	69	12.7	72	8.3	4	4.3	0.014 *
Peer problems	294	14.2	102	18.8	159	18.3	19	20.4	0.005 *
Prosocial behavior	89	4.3	29	5.3	29	3.3	4	4.3	0.341
Total Difficulties	259	12.5	105	19.3	121	13.9	12	12.9	0.001 *

* *p* value is significant at 0.05 or less.

**Table 5:** Behavioral and emotional problems among adolescents according to nationality.

Subscale/ Scale	Adolescents (12–18 Years) N = 4400										
	Jordanian n = 2912		Syrian Camp Refugees n = 509		Syrian Urban Refugees n = 738		Palestinian Camp Refugees n = 76		Others n = 165		*p* Value
	n	%	n	%	n	%	n	%	n	%	
Emotional problems	415	14.3	71	13.9	97	13.1	5	6.6	33	20.0	0.064
Conduct problems	511	17.5	113	22.2	111	15.0	13	17.1	27	16.4	0.027 *
Hyperactivity	371	12.7	50	9.8	85	11.5	8	10.5	26	15.8	0.207
Peer problems	457	15.7	98	19.3	115	15.6	14	18.4	31	18.8	0.256
Prosocial behavior	313	10.7	63	12.4	94	12.7	6	7.9	16	9.7	0.370
Total Difficulties	567	19.5	113	22.2	131	17.8	14	18.4	41	24.8	0.151

* *p* value is significant at 0.05 or less.

**Table 6:** Factors associated with total difficulties among children in binary logistic regression analysis.

Variable	Category	Reference	Odds Ratio	95% Confidence Interval		*p*-Value
				Lower	Upper	
Gender	Male	Female	1.445	1.151	1.815	0.002 *
Region	Middle	North	1.060	0.805	1.395	0.680
	South	North	1.175	0.825	1.675	0.371
Nationality	Syrian living in a camp	Jordanian	1.811	1.221	2.686	0.003 *
	Syrian living outside camps	Jordanian	0.862	0.634	1.171	0.343
	Palestinian living in a camp	Jordanian	1.211	0.596	2.461	0.597
Number of traumatic events	1	0.0	1.124	0.771	1.637	0.543
	2	0.0	1.466	0.988	2.176	0.057
	3	0.0	2.682	1.819	3.956	0.000 *
	≥4	0.0	5.353	3.810	7.523	0.000 *
Fruit intake (serving/day)	0	≥3	1.540	0.942	2.518	0.085
	1–2	≥3	1.057	0.736	1.517	0.766
Vegetables (serving/day)	0	≥3	1.613	0.932	2.792	0.087
	1–2	≥3	1.021	0.706	1.477	0.911
Light physical activity (days/week)	0.0	≥5	1.726	1.035	2.878	0.036 *
	1–4	≥5	1.243	0.758	2.039	0.388
Problematic internet use score	At risk	Not at risk	1.980	1.575	2.490	0.000 *
Smoking	Yes (cigarettes or water pipe)	No	1.473	0.477	4.543	0.501
Family affluence	Low	High	2.042	1.185	3.518	0.010 *
	Medium	High	1.597	0.968	2.634	0.067

* *p* value is significant at 0.05 or less.

**Table 7:** Factors associated with total difficulties among adolescents in binary logistic regression analysis.

Variable	Category	Reference	Odds Ratio	95% Confidence Interval		*p*-Value
				Lower	Upper	
Gender	Male	Female	0.870	0.722	1.049	0.144
Region	Middle	North	1.307	1.044	1.636	0.019 *
	South	North	1.376	0.997	1.899	0.052
Nationality	Syrian living in a camp	Jordanian	1.182	0.838	1.666	0.340
	Syrian living outside camps	Jordanian	0.843	0.640	1.109	0.222
	Palestinian living in a camp	Jordanian	0.890	0.452	1.750	0.735
	Other	Jordanian	1.375	0.883	2.141	0.159
Number of traumatic events	1	0.0	0.830	0.545	1.264	0.384
	2	0.0	0.847	0.561	1.280	0.431
	3	0.0	1.021	0.679	1.537	0.919
	≥4	0.0	2.260	1.619	3.156	0.000 *
Fruit intake (serving/day)	0	≥3	1.899	1.351	2.670	0.000 *
	1–2	≥3	1.049	0.809	1.362	0.717
Vegetables (serving/day)	0	≥3	1.519	1.077	2.144	0.017 *
	1–2	≥3	1.048	0.822	1.338	0.704
Light physical activity (days/week)	0.0	≥5	1.521	1.038	2.231	0.032 *
	1–4	≥5	1.117	0.766	1.630	0.564
Problematic internet use score	At risk	Not at risk	2.084	1.728	2.512	0.000 *
Smoking	Yes (cigarettes or water pipe)	No	1.447	1.176	1.781	0.000 *
Family affluence	Low	High	1.739	1.267	2.386	0.001 *
	Medium	High	1.256	1.005	1.571	0.045 *

* *p* value is significant at 0.05 or less.

## Data Availability

The datasets generated during and/or analyzed during the current study are available from the corresponding author upon reasonable request.
